# A New Paradigm to Indicate Antidepressant Treatments

**DOI:** 10.3390/ph14121288

**Published:** 2021-12-10

**Authors:** Anton J. M. Loonen, Taichi Ochi, Lisanne M. Geers, German G. Simutkin, Nikolay A. Bokhan, Daniël J. Touw, Bob Wilffert, Alexander N. Kornetov, Svetlana A. Ivanova

**Affiliations:** 1 PharmacoTherapy, Epidemiology and Economics, Groningen Research Institute of Pharmacy, University of Groningen, Antonius Deusinglaan 1, 9713 AV Groningen, The Netherlands; t.ochi@rug.nl; 2 Department of Clinical Pharmacy and Pharmacology, University Medical Center of Groningen, University of Groningen, Hanzeplein 1, 9713 GZ Groningen, The Netherlands; l.m.geers@umcg.nl (L.M.G.); d.j.touw@umcg.nl (D.J.T.); 3 Rijnstate Ziekenhuis, Hospital Pharmacy, Wagnerlaan 55, 6815 AD Arnhem, The Netherlands; 4 Mental Health Research Institute, Tomsk National Research Medical Center of the Russian Academy of Sciences, 634014 Tomsk, Russia; ggsimutkin@gmail.com (G.G.S.); bna909@gmail.com (N.A.B.); ivanovaniipz@gmail.com (S.A.I.); 5 Department of Psychiatry, Addictology and Psychotherapy, Siberian State Medical University, 634050 Tomsk, Russia; 6 Department of Psychotherapy and Psychological Counseling, National Research Tomsk State University, 634050 Tomsk, Russia; 7 Pharmaceutical Analysis, Groningen Research Institute of Pharmacy, University of Groningen, Antonius Deusinglaan 1, 9713 AV Groningen, The Netherlands; 8 Department of Fundamental Psychology and Behavioral Medicine, Siberian State Medical University, 634050 Tomsk, Russia; alkornetov@gmail.com; 9 Division for Control and Diagnostics, School of Non-Destructive Testing and Security, National Research Tomsk Polytechnic University, 634050 Tomsk, Russia

**Keywords:** depression, antidepressants, mood disorders, forebrain, neural circuits, natural resilience, placebo, treatment, habenula

## Abstract

This article develops the idea that clinical depression can be seen as a typical human response, largely rooted in human culture, to events of loss or times of adversity. Various biological, psychological, and social factors may cause some individuals to have a depressive reaction that is ineffectually limited in time and/or severity. Recovery occurs mainly based on natural resilience mechanisms, which come into play spontaneously, but which are sometimes inhibited or blocked by specific pathological biopsychosocial mechanisms. One of the mechanisms for this could be the influence of the circuits that regulate pleasure and happiness, along the dorsal diencephalic connection (DDC) pathway from the forebrain to the midbrain via the habenula. Therapy works by undermining the biopsychosocial factors that prevent the natural recovery mechanism from working. Treatment should, therefore, be seen as facilitating rather than causing natural recovery. This approach is in line with the high recovery rate after placebo treatments and the positive influence of pharmacological treatments with completely different sites of action. Acceptance of this model means that when studying new treatments for depression, a new paradigm must be applied in which the relative value of antidepressant treatment is specifically weighted in terms of enabling the natural resilience process.

## 1. Introduction

Since the pioneering ideas of Emil Kraepelin [1], neuroscientific psychiatry has been dominated by the paradigm that psychopathologies, such as schizophrenia and bipolar disorder, can be considered as ‘nosological entities’: well-defined disease units, which can be mutually distinguished [2,3,4,5]. This starting point still determines the search for genetic or biochemical factors which are pathophysiologically related to these disorders and may have predictive value in their course and choice of specific treatments. It is also the implicit starting point of the current systems for classifying mental disorders in clinical practice and research: the most recent edition of the Diagnostic and Statistical Manual of Mental Disorders (DSM-5) of the American Psychiatric Association [6], as well as the International Classification of Diseases (ICD-11) of the World Health Organization [7]. The reason for this conservatism may be that the dominant classification systems for mental disorders are of major importance for epidemiological researchers and health authorities. Moreover, the classical view that mental disorders are disease entities is supported by drug registration authorities all over the world by their policy that new drugs should be effective when treating patients with such defined disorders. In reality, randomized clinical trials on the efficacy of newly developed psychotropic drugs generally have rather disappointing results. Complete remission of the mental disorder is usually not the primary outcome criterion in such ‘pharma’-trials, although obviously, that would be the most relevant treatment objective. The reason for ignoring this primary goal of pharmacotherapy in pivotal controlled clinical trials might be the limited effectiveness of the tested drug in this regard.

This is particularly true for new antidepressant drugs when they are tested in people with at least moderately severe major depressive disorder (MDD), according to DSM criteria. A meta-analysis of 11 trials of the efficacy of vortioxetine showed, for example, that the complete remission rate differed by less than 10% between verum and the placebo [8]. Furthermore, less than one third of the verum-treated patients completely recovered within six to eight weeks [8]. It should be noted that the recovery rate of two other selective serotonin reuptake inhibitors (SSRIs), citalopram and paroxetine, was roughly the same in two earlier trials conducted by the Danish University Antidepressant Group (DUAG) and was considerably smaller than that of clomipramine [9,10]. Unfortunately, recent comparative trials of the remission rate with clomipramine as a treatment of major depression (according to the relatively broad DSM-IV or DSM-5 criteria) are missing in the scientific literature. We do not know what the remission rate would be if less strict criteria were applied than those as in the aforementioned studies by the DUAG. In the current article, we argue that the explanation for the low effectiveness of drugs in treating major depressive disorder in pharma-trials is mainly related to dominant social and cultural factors, which accompany the clinical picture in the generally accepted traditional notion. We attach great importance to the suggestion of Stassen et al. [11] that full recovery from a depressive episode is mainly related to the action of a natural resilience-like process that, in our opinion, is mainly empowered by the sociocultural motives of the individual. We conclude with a proposal to change the paradigm of antidepressant efficacy research and provide a theoretical example of this. We will start with a description of a few of our findings that brought us to this idea.

## 2. No Evidence for Specific (‘True’) and Nonspecific Antidepressant Drug Response

We recently studied possible associations between the responses to antidepressant treatment and specific pharmacogenes in over 150 antidepressant-free patients with depression [12,13]. These newly admitted patients had not been treated with antidepressant drugs during the preceding six months and 54.5% had never been treated with antidepressant drugs during their entire life. They had a clinical diagnosis of depression according to ICD-10 criteria [14] of at least moderate severity, as measured by Hamilton’s depression rating scale (HAMD-17) [15,16], and were studied over four weeks. According to the old but still very influential theory of Quitkin et al. [17], activation of the ‘true’ antidepressant mechanism takes 2–4 weeks to occur after antidepressant therapy starts, the initial treatment response being comparable with that of the placebo, hence nonspecific or spontaneous. The existence of such a lag time indicates that the acute pharmacological effects occurring after a short time result in activation of a unified mechanism that alleviates the complete syndrome. With this theory in mind, we compared the response during the first two weeks with that during the last two weeks. For the total group, the average HAMD-17 score amounted to 24.3 ± 5.2 (mean ± standard deviation) at entry, and this decreased to 12.9 ± 4.9 and 5.0 ± 3.9 after treatment at two weeks and four weeks, respectively [12]. Obviously, in this study, there was no question of perceiving a delayed response of two to four weeks for the antidepressants to show an effect in the depressed patients. The indication of the existence of such a ‘lag time’ through clinical experience has been debated by several authors when considering the course of the response over time in different patient groups in meta-analysis of controlled clinical trials [11]. The results of the research group of the Zürich Psychiatric University Hospital are particularly convincing in this respect [11]. These authors studied the individual pattern of improvement in 2848 patients with MDD who had participated in four independent clinical trials using a total of seven different antidepressants and a placebo. They found that the period to the onset of improvement (the latency time) and the pattern of improvement did not differ between the verum treatment and the placebo; however, the number of responders (incidence of improvement) was higher with verum than with the placebo. The fact that the early and large response in Ochi et al.’s study [12] was related to a placebo effect (a spontaneous resilience mechanism) was contradicted by the results of another study on the same population by our research group, which investigated the influence of polymorphisms of the gene encoding for P-glycoprotein (*ABCB1*) on the timing of the observed improvement [13]. Certain genotypes caused a partial shift in the improvement during the first two weeks compared to the second two weeks of treatment. This indicates that a pharmacological effect may at least contribute to the improvement in the clinical condition. The transporter P-glycoprotein limits the passage of antidepressants through the blood–brain barrier, and certain genetic variants may influence the rapidity and intensity with which CNS structures are pharmacologically affected by antidepressants. Of note, Stassen et al. [11] suggested that antidepressants activate ‘a common, biological, ‘resilience’-like component that largely controls recovery from depression’.

We suggest that the lack of a delayed specific antidepressant effect in our study is more related to the current types of depression covered by major depressive disorder in comparison to former ‘endogenous’ depression than to the effects of antidepressants, therewith also following the idea of Stassen et al. [11] about the nature of the antidepressant mechanism of action.

## 3. What Could Be the Explanation for These Observations?

The above findings indicate that in the contemporary treatment of patients with depression, there does not seem to be any room left for a unique antidepressant effect that only occurs after a few weeks. Most of the improvement occurs very quickly and is consistent with a nonspecific antidepressant effect, as theorized by Quitkin et al. [17]. Yet, this nonspecific effect does not rest solely on a suggestion or spontaneous recovery; a pharmacological effect plays at least a role in initiating the recovery process. We, therefore, believe that a biopsychosocial factor keeps the individual in a depressed behavioral state. When this factor is attenuated by pharmacotherapy, psychotherapy, or spontaneously, a natural resilience mechanism can bring about a full recovery. In theory, this pathogenic factor can be fed continuously or repeatedly, requiring maintenance treatment. In the past, Anton J.M. Loonen and Svetlana A. Ivanova wrote extensively about the nature of the biopsychosocial factors that play a role in depression [18,19]. We will briefly summarize this below. We consider, in more detail, the idea that the natural resilience process is mainly related to the sociocultural components of depressive disorders. This has a direct relationship with how the concept of depression has been shaped throughout history and especially in the last two centuries.

### 3.1. A Theory of the Background Biopsychosocial Components

**Figure 1:**
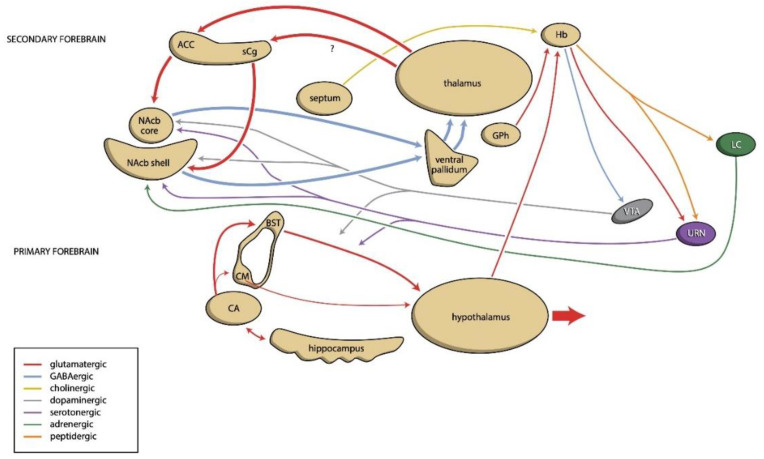
Hypothetical model representing the connectivity of the human primary and secondary forebrain. Our hypothesis suggests that the emotional response is initiated by the amygdalo-hippocampal complex and executed by the hypothalamus. The intensity of reward-seeking and distress-avoiding behavior is regulated by two parallel sets of cortical-striatal-thalamic-cortical (CSTC) circuits within the secondary forebrain, which include the nucleus accumbens (ventral striatum). The habenula controls the activity of these circuits by affecting ascending monoaminergic neuronal pathways. For further substantiation of this model, see Loonen and Ivanova [18,19]. ACC—anterior cingulate cortex; BST—bed nucleus of the stria terminalis; CA—corticoid part of the amygdala; sCG—subgenual cingulate cortex; CM—centromedial nucleus of the amygdala; GPh—the human equivalent of the habenula-projecting part of the globus pallidus; LC—locus coeruleus; Hb—habenula; NAcb—nucleus accumbens; URN—upper raphe nuclei; VTA—ventral tegmental area; CM and BST—extended amygdala.

Theory of knowledge teaches that scientific endeavor needs a model: a simplified representation of reality along the lines of a certain theory, suitable to effectively create a hypothesis that can be experimentally falsified or preliminarily confirmed. A fruitful method to combine the relevant neurochemical and neuroanatomical components into a single theory involves simplifying these structures of the central nervous system according to their evolutionary genesis. In line with this proposal, we developed a model as shown in Figure 1. Here, the primary forebrain, which regulates the essential behaviors associated with feeding, defending, and reproducing, was already present in the very first vertebrates (living 560 million years ago (mya)) and is represented in humans by the amygdaloid and hippocampal complexes and the hypothalamus. The secondary forebrain was already found in early amphibians (living 370 mya), and in humans, it consists of limbic ventral extrapyramidal circuits that regulate that willingness and intensity of these essential behaviors. The activity of the primary and secondary forebrain is regulated by ascending dopaminergic, serotonergic, and adrenergic pathways from the midbrain, which, in turn, are controlled by the dorsal diencephalic connection system (habenula).

By integrating physiological and pharmacological information with this anatomical model, we came up with the idea of the existence of two sets of limbic extrapyramidal re-entry circuits (see Figure 1) that partially independently drive the intensity of reward-seeking or distress-avoiding behavior, which, when successful, result in pleasure or happiness, respectively. This corresponds to the existence of two interrelated components of depressive disorders: one regulating the energy level and appetitive motivation and the other related to worrying and feelings of hopelessness. This results in a theoretical model where primary parts of the forebrain still initiate the emotional response when an opportunity occurs to obtain food, or to mate, or when safety is endangered. The secondary part of the forebrain, which regulates the readiness to, and the intensity of, the generated emotional response, is probably directly involved in addiction and bipolar disorder, as well as in depressive disorders. This model can also be applied to explain the rapid antidepressant effect of (es)ketamine. This substance probably acts on the habenula, regulating the activity of the ascending monoaminergic pathways from the midbrain.

The application of this model could be useful in defining psychopharmacological effects with a potential benefit in patients with mood disorders. Mood disorders are probably best considered to consist of a variable set of regulatory dysfunctions, which are neither unique for a specific type of mood disorder, nor essential for any one of them. Such dysfunctions could be defined within the context of the model to identify possible goals of treatment. Effective treatments of the largest disease-contributing dysfunctions may already enable natural resilience mechanisms to induce further (partial or complete) recovery. Therefore, once essential contributing biological factors allow for it, the sociocultural mechanisms can sufficiently motivate the individual to abandon depressive behavior. As the effect of natural resilience mechanisms, which also occur when the individual is treated with a placebo [11], is minimalized in pharma-trials, the effectiveness of antidepressant drugs is probably artificially lowered.

### 3.2. The Backgrounds of the Resilience Component

In addition to the above biological evolution that took more than 500 million years, humans also culturally evolved during fewer than the last 50,000 years [20]. Essential to the development of human culture and technology was the acquisition of writing skills. Writing enabled humans to communicate without direct physical contact, which thereby facilitated developments that built on the ideas of others. This skill has also played a major role in developing our thinking about having depression. Throughout about three millennia, the opinions of the consecutive writers on the symptomatology and impact on quality of life concerning mood disorders have been largely preserved. This facilitates the portrayal of the concept of depression as not being a recent psychiatric discovery, but as part of human history. The writings of ancient Israelian prophets [21] and Greek philosophers [22] still partly determine our views concerning mental illnesses. Their viewpoints were included in medieval Christian writings, where certain depressive states were also considered a mortal sin, particularly by Gregory the Great (Pope 590–604 AD) [23]. The influence of Genghis Khan (1162–1227AD), who expanded his Mongol Empire across Eurasia, thus allowing the views of Chinese, Indian, and Persian natural philosophers to be freely exchanged, should also be considered, especially in the more eastern parts of Europe [24]. ‘His empire eventually encompassed all or part of modern China, Mongolia, Russia, Ukraine, Korea, Azerbaijan, Armenia, Georgia, Iraq, Iran, Kazakhstan, Kyrgyzstan, Uzbekistan, Tajikistan, Afghanistan, Turkmenistan, Moldova, Kuwait, Poland, and Hungary’ [24]. The religious beliefs that found their basis in medieval thought eventually determined the type of madness that nineteenth-century brain psychiatrists engaged with [25]. This undeniably also applies to the disease categories described by Emil Kraepelin [1]. He began by studying the syndromes in patients in Estonian psychiatric asylums [26,27]. This means that he excluded many mentally disturbed patients who were considered more sinful or bad than mad, and/or when they belonged to the higher social classes. The same is probably true for other nineteenth-century psychiatrists [4,26,27,28]. In addition, Kraepelin himself did not speak Estonian [29] and his findings were colored even further by the pre-scientific views of the interpreters involved. The main point we want to make is that leading psychiatrists of the nineteenth and early twentieth centuries did not escape from the dominant religious beliefs of their societies that considered that depression could be madness as well as sinful/bad behavior or character weakness. They excluded several mood disorders from consideration because these were considered to belong to the religious and/or juridical domain; in that sense, their selection was also based on the prejudices fed by historical writings and popular pre-scientific beliefs about psychiatric illnesses. However, this has been ignored by late twentieth-century psychiatrists, who have ‘atheoretically’ included these depressive human problems without restrictions in the final MDD disease categories of the DSM-5 [6] and ICD-11 [7].

Why is this important for the purposes of this article? We have argued that depression should, to a large extent, be seen as a form of reaction to stressful sociocultural circumstances. We would like to add here that sociocultural factors are also a major independent driving force behind the natural resilience mechanisms that are so important for recovery from MDD. In other words, these mechanisms are likely a result of the pressure felt by the individual patient to meet the requirements put to them as a ‘good’ member of society.

### 3.3. The Relative Role of Biopsychosocial and Sociocultural Mechanisms

Based on the above, we would like to suggest that biopsychosocial mechanisms only keep people depressed and may facilitate their becoming depressed. These mechanisms are likely mainly related to primary and/or secondary forebrain functioning and may be targets of antidepressant treatment. When the influence of these mechanisms is weakened, due to treatment or spontaneously, natural resilience mechanisms can lead to recovery. These mechanisms are mainly related to the social and cultural obligations of every member of society, which therefore refers to the sociocultural component of depression.

We want to emphasize that different biopsychosocial factors can play a role and that the effect of a given treatment can vary. The same biopsychosocial factor can also play a role in different syndromes. The more they bear a succinct (and unique) character in a patient with depression, the more difficult it becomes to recover due to the natural resilience process; this makes recovery from bipolar or former ‘endogenous’ depression more difficult than from major depressive disorder according to DSM-5 [6] criteria. The effect of different treatment methods can, of course, also differ per individual. This explains why some patients do not respond to treatment with an SSRI or a tricyclic antidepressant but then recover by lithium addition, tranylcypromine, or ketamine.

## 4. Discussion

In summary, fruitful leads to alternative psychopharmacological mechanisms, and biomarkers suitable to magnify their impact could be derived by considering the evolutionary genesis of the forebrain and its implications for the interaction between neuronal systems. Depression describes a behavioral reaction form with a complex mechanistic background. Altered activity of circuits regulating pleasure and happiness is probably involved, but several different pharmacological (and psychological) mechanisms may accomplish these changes, and such altered activity has a role in several mental disorders. In order to maximize the treatment response of individuals, we should be able to identify people (by applying biomarkers) in whom the pharmacological effect induces the largest change in function. For example, the antidepressant response is related to the stimulation of the habenular dopamine D4 receptor (DRD4) by adrenergic fibers. Therefore, a clinical pharmacological study using neuropsychological instrumentation could examine whether the response of an adrenergic-acting antidepressant (e.g., reboxetine) depends on a functional polymorphism of *DRD4*. If the response is positive, pragmatic studies can then determine whether people with the associated polymorphism demonstrate a better response after treatment with an adrenergic-acting antidepressant than those without genotyping. Hence, the new paradigm of functional psychopharmacology includes three major components:

1. Specify and apply psychopharmacological effects that can induce a relevant change in neuronal forebrain functioning;
2. Identify individuals in which the induced change is optimal;
3. Investigate and verify in comparative pragmatic studies whether the change is sufficient to allow further recovery due to sociocultural mechanisms recruiting natural resilience mechanisms.

## 5. Conclusions

A paradigm change might be necessary for the successful treatment of commonly observed mood disorders. As depressive disorders are partly determined by sociocultural processes, which allow the individual to recover by recruitment of natural resilience mechanisms, psychopharmacological treatment effects are not necessarily related to full recovery. A partial response may be enough to initiate a resilience mechanism that enables the individual to abandon depressive behavior. Evidence for the existence of clinically relevant treatment effects can be obtained in clinical psychopharmacological experiments in which the influence of the natural resilience mechanism is minimalized. The usefulness of the treatment effects should, thereafter, be estimated in comparative pragmatic studies. In our opinion, the registration authorities would be advantaged by modernizing their research guidelines equally.

## Data Availability

Data sharing not applicable.
